# Something to Snack on: Can Dietary Modulators Boost Mind and Body?

**DOI:** 10.3390/nu15061356

**Published:** 2023-03-10

**Authors:** Mathilde C. C. Guillaumin, Boris Syarov, Denis Burdakov, Daria Peleg-Raibstein

**Affiliations:** Institute for Neuroscience, Department of Health Sciences and Technology, ETH Zürich, 8603 Schwerzenbach, Switzerland

**Keywords:** nutrition, macronutrients, dietary modulators, cognition, motivation, exercise, memory, learning, mice

## Abstract

The last decades have shown that maintaining a healthy and balanced diet can support brain integrity and functionality, while an inadequate diet can compromise it. However, still little is known about the effects and utility of so-called healthy snacks or drinks and their immediate short-term effects on cognition and physical performance. Here, we prepared dietary modulators comprising the essential macronutrients at different ratios and a controlled balanced dietary modulator. We assessed, in healthy adult mice, the short-term effects of these modulators when consumed shortly prior to tests with different cognitive and physical demands. A high-fat dietary modulator sustained increased motivation compared to a carbohydrate-rich dietary modulator (*p* = 0.041) which had a diminishing effect on motivation (*p* = 0.018). In contrast, a high-carbohydrate modulator had an initial beneficial effect on cognitive flexibility (*p* = 0.031). No apparent effects of any of the dietary modulators were observed on physical exercise. There is increasing public demand for acute cognitive and motor function enhancers that can improve mental and intellectual performance in daily life, such as in the workplace, studies, or sports activities. Our findings suggest such enhancers should be tailored to the cognitive demand of the task undertaken, as different dietary modulators will have distinct effects when consumed shortly prior to the task.

## 1. Introduction

Food is classically perceived as a means to provide energy and building material to the body. In the past decades, it has become increasingly recognized that food has the ability to prevent and protect against various diseases [1]. This accumulated knowledge demonstrates that food can have a broad impact (positive as well as negative) on a range of molecular systems supporting neuronal function and plasticity. Until now, pharmacological cognitive enhancers have been used primarily to treat patients with cognitive difficulties, such as Alzheimer’s disease, Parkinson’s disease, and attention-deficit hyperactivity disorder [2]. However, interventions that aim to improve cognitive performance beyond what is necessary to sustain good health are also of interest to the general public. Although many recent studies emphasize the important effects of food on the brain, further work is necessary to determine causal links to cognitive performance and, crucially, the time windows when incorporating specific food components into the diet can improve cognition. During the last two decades [3], there were significant advances in elucidating the role of diet in improving cognitive and mental performance beyond merely exercise and physical performance. Historically, the targeted consumers were mainly athletes and bodybuilders, and the functional foods were sports nutrition products [4]. With the increase in consumer health consciousness, the interest in these functional foods expanded to recreational and lifestyle users [5]. Due to increased consumer interest in these foods and beverages, nutrition research shifted its direction to identify nutrients that support energy metabolism, improve overall performance, and increase muscle mass as well as physical performance [6,7]. This led to the development of functional foods and drinks that are sold in every gas station, supermarket, kiosk, and drug store. These products are advertised as a help, for example, to support exercise load, reduce exercise fatigue, or improve recovery following an intense workout. Due to the growing popularity of these functional foods, there is now increasing interest and demand in shifting their development towards the enhancement of behavioral and psychological functions. The next step will be to design targeted foods/beverages (from now on referred to as “dietary modulators”), which are tailored to the type of cognitive task, subjective mental state, gender, age group, and sleep-wake state.

In principle, dietary modulators for behavioral and psychological responses could be directly identified, but the complexity of these responses makes this identification far from simple. There is also a clear need to distinguish between short-term postprandial responses to food ingestion and long-term effects of dietary adjustment, which requires different research methodologies. Therefore, research related to the cognitive and motivational effects of dietary supplements needs sensitive psychobiological assays. Thus, the aim of this study was to identify the short-term effects of the consumption of a certain dietary modulator—in the form of a snack—on specific cognitive functions, such as learning, memory, cognitive flexibility, motivation, and motor function. We tested four different dietary modulators: a control (balanced macronutrient composition), carbohydrate-, protein- or fat-rich snack. These modulators were similar in volume and sensory properties (taste, consistency, and color) and were given 20 min prior to testing. For cognitive evaluations, we employed automated, touchscreen-based operant chambers which allowed us to assess multiple cognitive domains within the same testing environment [8]. We chose a battery of tests comprising highly translatable paradigms to assess a range of cognitive abilities. This included a visual discrimination task to estimate cognitive flexibility, a progressive ratio task to evaluate motivation, and voluntary physical activity (wheel running) to measure motor function. The selected tests represent single components that form part of more complex skills and abilities (i.e., driving a car, ability to operate machinery, writing a test, etc.). Testing was performed within the same animals following different dietary modulators.

## 2. Materials and Methods

### 2.1. Subjects

A total of 9 C57BL/6J mice were kept on standard chow (3430 Kliba Nafag, Kaiseraugst, Switzerland) and water ad libitum and on a reversed 12 h/12 h light/dark cycle. Experiments were performed during the dark phase. Adult mice (at least 8-week old), all group-housed, were used for the experiments. Prior to starting behavioral testing, water bottles were switched to 2% citric acid water. This water regime was shown to have only subtle impacts on willingness to perform behavioral tasks utilizing plain water as a reward [9,10,11]. 

### 2.2. Feeding Schedule and Preparation of Dietary Modulators

The protein and carbohydrate dietary modulators were adapted from [12], the fat modulator was adapted from Kliba 60% high-fat diet (2127 Kliba Nafag, Kaiseraugst, Switzerland), and the control diet replicated the normal laboratory chow (3430 Kliba Nafag, Kaiseraugst, Switzerland). All four dietary modulators were matched for micronutrient composition, and the ingredients used were kept the same with only proportions varying to account for the different macronutrient levels of each modulator. Modulators were prepared in-house and were designed in accordance with the information on the EFSA and FDA websites regarding the definition of high-fat, high-protein, and high-carbohydrate diets. Mice received the exact amount of food calculated for each dietary modulator so that the caloric intake for all of them was kept strictly the same. All animals were exposed to each modulator, but the order in which they were exposed to the different modulators was randomly assigned (using https://www.randomizer.org, accessed on 1 September 2020). This means that for each behavioral task, each mouse underwent four blocks of testing (one block per modulator). A block consisted of at least 5 days, during which the mice received the modulator once daily prior to the behavioral task to be performed on that day (mice had access to normal chow in their home cage during the rest of the day). Between the different dietary modulators, there was a two-day wash-out period during which all mice were only given their normal laboratory chow (i.e., no additional snack on those two days). This was necessary to rule out any potential transfer between the different dietary modulators consumed. All dietary modulators had similar taste, smell, and texture.

### 2.3. Compositions

Proteins, carbohydrates, and fats were the three main macronutrient components in each dietary modulator, as some of the largest differences in one’s diet derive from changes in those three macronutrients. Consequently, the three modulators were prepared as follows: high-fat (‘Fat’) with 60% of calories coming from fat, high-protein (‘Protein’) with 50% of calories coming from proteins, high-carbohydrate (‘Carbohydrate’) with 80% of calories coming from carbohydrates, and a control modulator (‘CTR’) (Table 1). The CTR modulator was prepared, mimicking the macronutrient composition of the normal laboratory chow diet (3430 Kliba Nafag, Kaiseraugst, Switzerland) to which mice had access in their home cage. All dietary modulator components were precisely measured, and the samples were kept in the freezer for the duration of the experiments. Before each meal, the modulators were taken out and let to come back to room temperature before being given to the animals.

### 2.4. Amounts

All mice were habituated in their home cage to the dietary modulators prior to the start of the experiments with a small amount of the food given for 2 consecutive days. Food was fully eaten after each day. For dietary modulator consumption prior to each experiment, animals were placed in a feeding station where each mouse had its own serving plate with the exact amount of dietary modulator. All animals were habituated for 3 days to this setup prior to the start of experiments to reduce neophobia and novelty-related stress. For each task (VD/rVD, PR, RW), 20 min prior to the daily experiment, the dietary modulator (0.425 g ± 0.075 g containing 1.015 kcal) was given in individual feeding stations, and animals were allowed to eat the food for 10 min, after which, animals were placed back into their home cages until the beginning of the session. Due to the randomization of the order in which mice received the modulator, on a given day, all mice did not receive the same modulator.

### 2.5. Cognitive Assays

We employed the touchscreen testing system (Campden Instruments Ltd., Loughborough, Leicestershire, UK), as it has several advantages over a classical operant testing apparatus, such as a high translational potential, similarity to psychological and cognitive tests used in human subjects, and lower stress levels for the animals. The cognitive assays we were interested in to test specific psychological domains all employed the same types of responses, reward (tap water), and stimuli (adapted from [13]). Eight mouse touchscreen chambers (Campden Instruments Ltd., Loughborough, Leicestershire, UK) were used for the visual discrimination (VD), fixed ratio (FR), and progressive ratio (PR) tasks. The behavioral programs were controlled by the ABET II Touch software (version 21.02.15.0, Campden Instruments Ltd., Loughborough, Leicestershire, UK) and the Whisker Server (version 4.7.7, Cambridge University Technical Services Ltd., Cambridge, UK, [14]). All animals were habituated to the chambers for 20 min over 2 consecutive days before testing commenced.

For the VD/reverse VD and FR/PR tasks, an individual criterion was used so that immediately after reaching the acquisition phase criterion, a mouse was moved on to the next phase to avoid overtraining. In practice, this means that instead of waiting until all animals reached criterion before proceeding to the primary task/test phase (which would have meant that some animals would be overtrained and have obtained more rewards), animals moved on to the next phase at their own pace. This individual criterion approach allowed us to avoid carry-over effects.

### 2.6. Visual Discrimination (VD) Task

The visual discrimination (VD) cognitive task (evaluating learning, cognitive flexibility, attention, and working memory) is based on the capability of a mouse to distinguish between two visual stimuli where one stimulus is reinforced (S+) and the second stimulus is not (S−). The S+ and S− were counterbalanced between animals.

#### 2.6.1. Training Phase

This phase was performed before the start of the dietary modulator schedule.

*Habituation*—Animals were introduced to the touchscreen chambers over 2 days with each session lasting 30 min or 50 trials (whichever came first) and were given a reward (tap water) every 5 s.

*Initial touch*—This stage lasted for 3 days with each session lasting either 30 min or until completion of 100 trials. Mice were given a reward every 30 s and were given the choice to touch a white square on the touchscreen, which resulted in receiving a reward 3 times larger.

*Must touch*—This stage lasted for 3 days with each session lasting either 30 min or completion of 100 trials. Here, mice had to actively poke the screen in order to receive a reward.

*Must initiate*—This stage lasted for 3 days with each session being either 60 min or completion of 100 trials. Here, mice needed to start each trial from the same position to avoid a location bias. The must-initiate step was activated by breaking the infrared beam in the food magazine, and the initiation was coupled with a sound so that the mice did not confuse it with reward collection.

*Punish incorrect stage*—This stage lasted for 3 days with each session being either 30 min or completion of 100 trials. Incorrect responses resulted in a time-out period of 10 s which was coupled with turning on the house light during the first 5 s. Once that time had passed, mice were allowed to initiate a new trial.

#### 2.6.2. Initial Acquisition Phase (VD/Reverse VD in the Absence of Dietary Modulators)

The mouse needed to distinguish between two visual stimuli where one stimulus was always reinforced (S+), and the second stimulus was never reinforced (S−). The S+ and S− were counterbalanced between animals. Animals needed to successfully reach criterion which was to achieve 80% correct responses. Once they achieved this stage, the contingency was reversed (the stimulus associated with reward became S− and vice-versa) until mice reached the criterion of 80% correct trials again.

Correction trials were introduced during the training and differed from ‘normal’ trials in that following an error (mouse pressed the S− stimulus), the previous stimulus was repeated (and not reassigned randomly) in the same location until the correct stimulus/location was selected. This step was important to prevent an animal from selecting one location by chance and being rewarded on 50% of the trials.

#### 2.6.3. VD and rVD under Dietary Modulators

Following the conclusion of the acquisition phase, all animals were trained on a series of VD and reverse VD learning experiments using new visual stimuli and exposing mice each time to a different dietary modulator. Similar to the acquisition phase, the criterion to move on from VD to rVD was to reach an 80% success rate within 20 days of training. If a mouse could not reach criterion by the 20th day, data from all 20 days were taken and analyzed. Once a mouse had achieved the 80% correct responses for both VD and rVD phases, they waited until all mice reached completion, or the 20 days had passed. For each dietary modulator, a novel pair of stimuli was introduced.

### 2.7. Progressive Ratio (PR) Task

The first phase of the task required an invariant number of responses (pokes) for a fixed quantity of reinforcer, i.e., a fixed ratio (FR) schedule. Later, a progressive ratio (PR) schedule was introduced where the response requirement incremented with each reinforcer earned. The PR schedule allows for measurement of reward strength [15], but it is also sensitive to detect reward magnitude, palatability, as well as reinforcer state [16,17]. Therefore, we employed the PR task to assess the motivational state of the mice in response to the different dietary modulators.

#### 2.7.1. Training Phase

The first four stages (habituation, initial touch, must touch, and must initiate) were the same as described for the VD task.

#### 2.7.2. Fixed Ratio Schedule (FR)

Animals were trained under FR1, FR2, FR3, and FR5. The amount of reward (tap water) stayed the same for all FRs; only the number of pokes required to obtain it changed. FR1, FR2, and FR3 were carried out for one session each, whereas FR5 was carried out over three sessions. Following the completion of the FR schedules, all animals started the PR schedule.

#### 2.7.3. Progressive Ratio Schedule (PR)

The PR protocol consisted of progressive increments of the number of pokes required in order to obtain the reward. A PR of 4 was chosen for the task; i.e., 1, 5, 9, 13, 17, 20, and so on, pokes were successively required to obtain one reward. Each mouse was given 5 days on each dietary modulator. After those 5 days, mice were kept with their normal diet with no addition of a modulator for 2 days before being introduced to the next dietary modulator. This served as a wash-out period and was important to eliminate residual effects of the previous modulator. The breakpoint was recorded after 90 min or if a mouse did not have any input (FM visit or screen poke) for 5 min. The breakpoint was defined as the last (largest) number of pokes necessary for the last reward received for each animal. This phase of the experiment was terminated after four weeks (4 dietary modulators).

### 2.8. Exercise Running Wheel Activity

Mice were given access to a low-profile running wheel (ENV-047, MED Associates/OpCoBe) for 2 h per day over 5 days. Before each session, mice received a dietary modulator (same dietary modulator over the 5 days) in the same manner as in the FR/PR and VD/rVD tasks. There was a 2-day wash-out period before this 5-day protocol was repeated with another dietary modulator. All mice, therefore, performed this protocol 4 times (once for each modulator).

Running activity was recorded wirelessly using a USB hub (DIG-807, Med Associates/OpCoBe) itself connected to the Running Wheel Manager Data Acquisition software (version 2.03, MED Associates, Fairfax, VT, USA) with the amount of running recorded in 1 min bins. Wheels were placed in standard IVC cages; mice were moved to these cages for their daily running session before being moved back to their own home cage at the end of each session. Mice had been habituated to the low-profile running wheels before the start of the protocol to ensure familiarity with running on these wheels.

### 2.9. Data Analysis

Statistical analysis was conducted using SPSS (version 28.0.0.0, SPSS Inc., Chicago, IL, USA) with significance set at *p* ≤ 0.05. For the visual discrimination and/or reversal tasks, the measures include trials completed, response accuracy (% correct trials), number of correction trials, session duration, response and reward latencies, response omission, and the number of sessions required on each training stage to reach criteria. The effects of dietary modulators on these measures were examined by independent or paired *t*-tests, ANOVA, or repeated measures (RM) ANOVA, where appropriate, with consideration for multiple comparisons (Bonferroni correction if more than two modulators are compared). For all datasets, the z-score method was used to detect outliers (none were detected).

## 3. Results

### 3.1. Visual Discrimination (VD) Task

**Figure 1:**
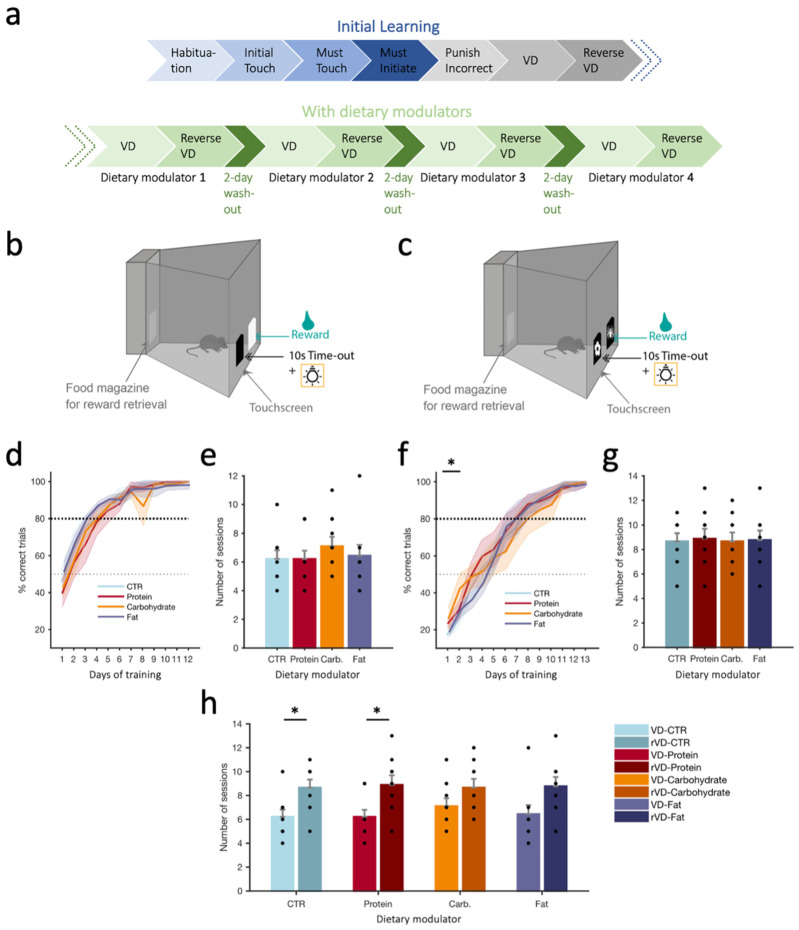
(**a**) Timeline of the visual discrimination (VD) and reverse VD (rVD) schedules during the learning phase (without dietary modulators) and under dietary modulators. (**b**) Schematic of the apparatus during the initial learning ‘Punish incorrect’ phase. (**c**) Schematic of the apparatus during the visual discrimination (VD) task (image pairs introduced). (**d**) Percentage of correct trials during the VD phase when all trials are considered under the different dietary modulators. (**e**) Number of sessions required to reach criterion for the VD phase under the different dietary modulators. Black dots indicate individual animals; animals with the same values overlap. (**f**) As in d, but for the reverse VD phase; asterisk refers to CTR vs. carbohydrate. (**g**) As in e, but for the reverse VD phase. (**h**) Numbers of sessions needed to reach criterion (>80% for 3 consecutive sessions) across dietary modulators in the VD and rVD phases. Values reported as mean ± SEM (**d**,**f**) or mean + SEM (**e**,**g**,**h**). N = 9 mice. *p*-values reported as follows: * *p* ≤ 0.05. Carb.: carbohydrate, CTR: control.

Here, we tested the acute effects of different dietary modulators on cognitive flexibility, which is a measure of higher cognitive abilities [18]. This type of behavioral flexibility can be studied experimentally by employing reversal learning tasks when subjects are required to adapt their responses to reversed reward contingencies. We were interested to identify whether short-term effects of certain dietary modulators would provide improvements for one phenomenon and not exert deleterious effects on others, as executive functions subserve the selection and processing of information necessary to plan, control, and direct behavior in a manner appropriate to changing environmental demands [19]. To evaluate the impact of the different dietary modulators on cognitive performance, we thus used a VD/reverse-VD (rVD) task (Figure 1a–c), allowing us to probe learning, cognitive flexibility, and working memory. Since most appetitive operant tests employ food as a reward, which, in our case, would confound assessing the effect of acute consumption of our dietary modulators, we used water as a reward instead. To avoid variation in the initial performance level during the acquisition of visual discrimination, each animal was trained to reach similar performance levels at the end of the training phase (performance criterion). This is important to assess alterations in performance in the reversal learning task due to the intake of the dietary modulator. When analyzing the accuracy of response, we found that the percentage of correct trials over sessions was not influenced by dietary modulators in the VD phase (Figure 1d). Additionally, the number of sessions needed to reach the performance criterion under each dietary modulator was used as a general measure of learning. We found a clear learning effect indicated by the main effect of the session for the percentage of correct trials (VD: F(11,88) = 131.44, *p* < 0.001, Figure 1d). The number of sessions to reach the criterion in the VD phase showed that under the carbohydrate modulator, animals seemed to need more sessions to reach the criterion, which would indicate difficulty in learning, but this did not attain significance (F(3,24) = 0.460, *p* = 0.750, Figure 1e). During the reversal learning phase (Figure 1f), the carbohydrate modulator led to increased performance during the first two sessions compared to the control (CTR) dietary modulator, which was made evident by a significant main effect of the dietary modulator when comparing those two modulators over the first two sessions (F(1,8) = 6.829, *p* = 0.031) in addition to the main effect of the session (F(1,8) = 10.413, *p* = 0.012). Pairwise comparisons showed a significantly higher percentage of correct trials for the carbohydrate vs. CTR modulator during the second session (*p* = 0.048; Figure 1f), indicating an initial beneficial impact of the carbohydrate modulator, leading to an initial increased performance that subsequently subsides. When further analyzing the general performance during reversal learning, the number of sessions needed to reach the performance criterion was highly similar across dietary modulators (F(3,24) = 0.021, *p* = 0.996, Figure 1g), although there was here again a clear learning effect with a significant main effect of the session (rVD: F(12,96) = 187.86, *p* < 0.001, Figure 1f). We further analyzed the differences in learning rates between acquisition and reversal phases across sessions (Figure 1h). RM ANOVA revealed a general performance difference between acquisition and reversal learning phases where all dietary modulators showed an increased number of sessions to reach the criterion during the reversal VD phase compared to the VD phase (F(1,8) = 32.40, *p* < 0.001). Pairwise comparisons indicated a significant difference in learning performance in CTR VD vs. rVD (*p* = 0.045) and protein VD vs. rVD (*p* = 0.05). These findings may indicate a cognitive flexibility deficit in the CTR and protein modulators. The fat and carbohydrate modulators were able to inhibit the prepotent response and instead allowed animals to learn the new contingency at a similar rate to the initial visual discrimination learning.

### 3.2. Progressive Ratio Schedule (PR)

**Figure 2:**
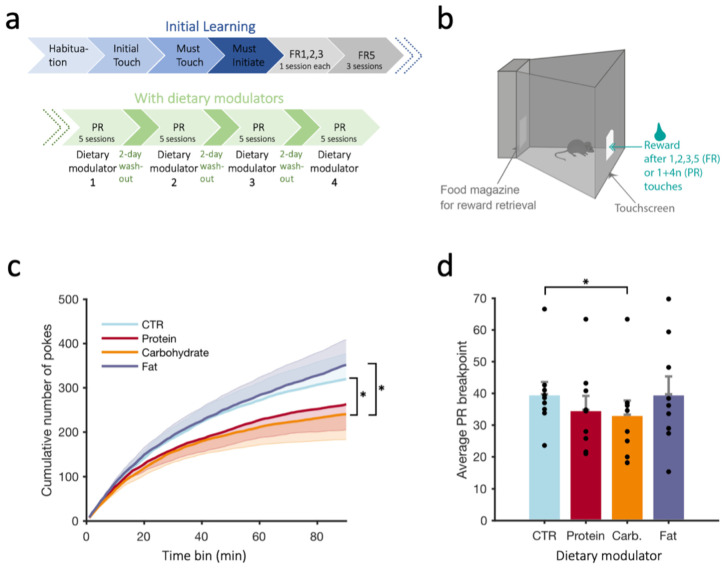
(**a**) Timeline of the habituation, fixed ratio (FR), and progressive ratio (PR) schedules during the learning phase (without dietary modulators) and under dietary modulators. (**b**) Schematic of the apparatus of the FR and PR tasks. (**c**) Cumulative number of pokes across a 90 min session for each dietary modulator. Each line represents the average over 5 sessions for each modulator. (**d**) PR breakpoint across the dietary modulators (average over 5 sessions for each modulator). Black dots indicate individual animals; animals with the same values overlap. Values reported as mean + SEM (mean-SEM for protein and carbohydrate in (**c**)). N = 9 mice. *p*-values reported as follows: * *p* ≤ 0.05. Carb: carbohydrate, CTR: control.

**Figure 3:**
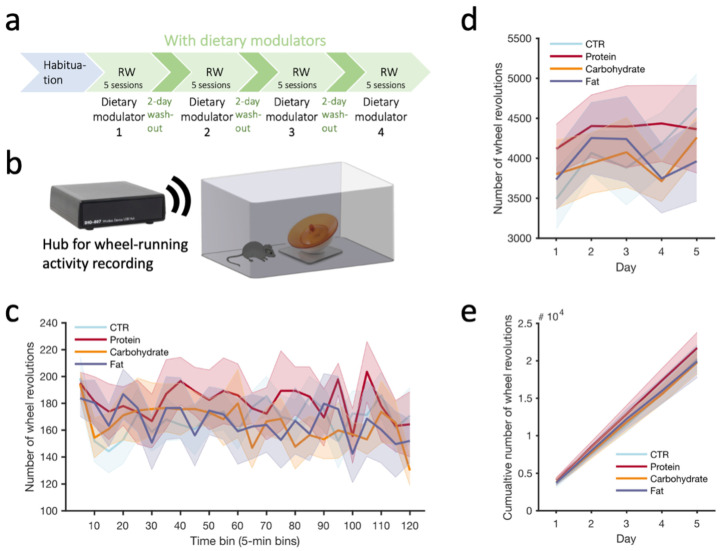
(**a**) Timeline of the habituation and running wheel (2 h daily sessions) under the different dietary modulators. (**b**) Schematic of the low-profile running wheel apparatus used during each session. (**c**) Running wheel activity (5-day average) across a 2 h session for each dietary modulator. (**d**) Running wheel activity (2 h sum) across days for each dietary modulator. (**e**) Running wheel activity cumulated across sessions (2 h sum) and days (5 sessions) for each modulator. # 10^4^ indicates the y-scale values are to be multiplied by 10^4^. Values reported as mean ± SEM. N = 9 mice.

We next evaluated motivation to work for a reward—and how this can be enhanced or impaired by a dietary modulator—using the PR task (Figure 2a). The number of pokes needed to receive one reward was incremented by four after each reward collection during the PR phase of the task (see Methods, Figure 2b). To evaluate how the performance of the mice on the PR schedule was influenced by dietary modulators, we plotted the pattern of pokes under this schedule as a cumulative distribution curve (Figure 2c). An RM ANOVA showed a significant main effect of the dietary modulator (F(3,24) = 2.938, *p* = 0.05). Furthermore, pair-wise comparisons revealed that the carbohydrate modulator significantly produced lower poke numbers compared to the control dietary modulator (*p* = 0.025). Additionally, the breakpoint is traditionally used as an indicator of motivation; an RM ANOVA of the mean breakpoint over five PR sessions (Figure 3d) indicated a significant main effect of the dietary modulator (F(3,24) = 2.938, *p* = 0.05). Pair-wise comparisons revealed a significant difference between the CTR and carbohydrate modulators (*p* = 0.018), indicating that the carbohydrate modulator significantly reduced motivation to work for a reward in this task. Such a negative effect was not observed for the protein and fat modulators. In addition, our data revealed a significant difference between fat and carbohydrates, whereby the carbohydrate modulator significantly reduced motivation compared to the fat modulator, as shown by the lower cumulative number of pokes (*p* = 0.041, Figure 2c).

### 3.3. Exercise Running Wheel Activity

Finally, to determine if different dietary modulators could alter physical performance, we recorded acute wheel running (Figure 3a,b) and analyzed running across the 5 2 h sessions completed for each modulator (see Methods, Figure 3c), looking at the mean running wheel activity (wheel revolutions) across 5 days (Figure 3d) as well as cumulative running across days (Figure 3e). We found that the different dietary modulators did not differentially influence the amount of wheel running either on a daily basis (Figure 3d) or if cumulating across all the sessions completed under a given modulator (Figure 3e). Additionally, one could hypothesize that the short-term effect of a given dietary modulator could alter the running activity only at the beginning (e.g., acute ‘boosting’ effect) or at the end (e.g., improves endurance) of a running session. However, we did not observe any significant effect for any of the modulators compared to the CTR (Figure 3c). It is interesting to note that the high-protein modulator yielded running levels that were always on the higher side for all three measures (Figure 3c–e), although this was not statistically significant.

## 4. Discussion

It is known that chronic or constant inadequate intake of macronutrients, such as carbohydrates, proteins, and fats, can compromise the optimal function of the human body. Accumulating evidence suggests that a diet balanced in macronutrient composition can have an important role to prevent diseases [20,21] and can also affect psychological and mental states [22,23,24,25]. However, the acute effects of a meal or snack with a specific macronutrient composition on performance are still largely unresolved, especially in the general population (beyond athletes). Yet, there is an increasing demand for acute cognitive and motor function enhancers to improve both mental/intellectual and physical activity demands in daily life, such as in work environments, during studying, or sports activities. Therefore, here, we were interested to assess how specific macronutrient compositions in the form of a dietary modulator consumed prior to cognitive testing or exercise could acutely affect cognitive and motor performances in healthy adult mice. A high-fat dietary modulator was found to induce increased motivation compared to a carbohydrate-rich dietary modulator, which had the opposite effect. However, the high-carbohydrate modulator gave an initial advantage in a cognitive flexibility task compared to the other modulators. None of the tested dietary modulators had any effect on learning in the initial visual discrimination phase. Finally, no apparent effects of the different dietary modulators were observed on physical exercise despite a trend for a positive influence on running, which was observed following a protein-rich dietary modulator.

Many rodent tests are fundamentally different from assessments used in human neuropsychological testing, which may be one of the reasons why preclinical findings often fail to translate to the clinical condition [26,27,28]. Therefore, in the present set of experiments, we used touchscreen operant boxes, which can assess complex cognitive abilities, such as discrimination learning, reversal learning, and motivation, similarly to human computer-based neuropsychological assessment tests [29,30,31]. Cognitive abilities can be often difficult to compare between species; however, an organism’s ability to adapt its behavioral repertoire to changing situations and environments is a translatable feature of cognition [32]. The visual discrimination learning task involves two processes: learning to discriminate between two stimuli and learning which of the two leads to a reward. In the initial visual discrimination acquisition performance phase, we did not observe any differences in learning between the different modulators. All animals were able to learn and perform the task properly and did not show any motivational decline. In reversal learning, the stimulus–reward contingency acquired during the initial discrimination phase is reversed, and animals need to inhibit the prepotent response to the previously correct stimulus and learn the new stimulus–reward contingency. Here, we detected initially enhanced learning in the carbohydrate group where animals performed significantly better across the first two reversal sessions and then evolved at a similar pace in the subsequent reversal sessions compared to the control modulator. A comparable behavioral profile was apparent when comparing the learning performance of each dietary modulator between the visual discrimination and reversal learning phases where animals needed more sessions to reach the performance criterion in the reversal phase; however, this difference was significant only in the control and protein modulator groups. This can indicate a general cognitive flexibility deficit in those two subgroups or enhanced flexibility with high-fat or high-carbohydrate modulators. In summary, because the visual discrimination phase performance did not differ between the different dietary modulators, the observed learning difference during the reversal phase cannot be attributed to impaired visual discrimination but is rather a consequence of deficits in cognitive flexibility in response to the control and protein modulators. In addition, the initial beneficial effect of the carbohydrate modulator on cognitive flexibility can suggest a possible role for carbohydrate consumption in early learning when the reversal was new but not in late learning when reversal learning was already familiar. This might be explained by the fact that ingestion of carbohydrate-rich foods produces only a short-lasting blood glucose spike since glucose is rapidly counter-regulated in the body and provides a ready source of energy. Studies in humans suggested that increased neural activity (e.g., the learning of a motor task and verbal working memory) was linked with increased use of glucose by the brain [33,34], which is consistent with the notion that cognitively demanding situations can deplete the brain of glucose [35,36].

When testing the acute and immediate effects of the different dietary modulators on motivation—again, utilizing water as a reward in the progressive ratio paradigm—the behavioral performance of all animals (as measured by a breakpoint) significantly increased during sessions (Figure 2c). However, intake of the carbohydrate modulator prior to testing significantly affected task performance with a decreased number of pokes and a decrease in average breakpoint (Figure 2d). In contrast, consumption of the fat modulator prior to testing significantly increased motivation relative to carbohydrate modulator consumption (Figure 2c). Importantly, the fat-mediated elevation in task performance did not appear to be the result of nonspecific locomotor hyperactivity, as no significant effects of fat modulator consumption affected wheel revolutions on the running wheel. Interestingly, a modulator high in carbohydrates improved cognitive flexibility in the early stage of a reversal-learning task (Figure 1f) but proved deleterious for a motivational task. Studies in the past decade have shown that certain diets consumed chronically over a long time period, where adaptation to a diet is being examined, can influence and maintain mental function. For example, diets enriched in omega-3 fatty acids (i.e., essential fatty acids) can enhance cognitive processes in humans [37,38,39,40] and promote restoration of brain homeostasis following brain injury in animals [39,41] by upregulating genes that are important for maintaining synaptic function and plasticity. In contrast, foods with a high content of both sugars and fats are known to negatively interfere with molecular substrates of cognitive processing and increase the risk of neurological dysfunctions [42,43] and produce changes in the hippocampus and negatively impact memory and learning [44,45,46,47,48]. Even a short period of a high-fat diet (a few days) was found to reduce working and episodic memory in adult humans [49]. However, the exact fatty-acid composition and ratios of other nutrients matter, as ketogenic diets, which are by definition high in fat, can have significant beneficial effects on cognition and some brain conditions, in particular, epilepsy [50,51,52]. What all studies seem to agree on and suggest is that poor dietary habits over longer time periods can lead to diverse negative health implications, including cognitive and mood dysfunctions. Here, we found that an acute intake of the modulator high in fat increased motivation. Our findings go in line with previous studies, among which, a study performed on young men who were given a fat-rich breakfast was found to positively influence different cognitive functions, which were assessed with the aid of computer tests for short-time memory, reaction time, and attentiveness [12].

The absence of effect of the high-protein modulator may appear surprising, as some amino acids are precursors of various neurotransmitters and neuromodulators (e.g., tryptophan is a precursor for serotonin and melatonin; tyrosine for dopamine, noradrenaline, or adrenaline) [53,54]. Thus, tyrosine, as a precursor of dopamine and noradrenaline, is involved in the regulation of attention, arousal, and motivation [55,56]. However, several factors need to be borne in mind. Amino acids must cross the blood–brain barrier to affect brain function, and for this, they require specific transporters for which amino acids can be in competition against one another [54]. Therefore, the link between the amino acid content of a meal and the subsequent levels of neurotransmitters in the brain is not straightforward. For example, a meal rich in tryptophan will not necessarily lead to an increase in serotonin; in fact, it was shown that a protein-rich meal decreased serotonin concentration, while a meal with a low protein:carbohydrate ratio increased those levels (as remaining tryptophan in the bloodstream no longer competes with other amino acids which are taken up by the muscles in response to insulin) [54,57,58]. In the case of glutamate, which is a neurotransmitter itself, the literature has shown that dietary glutamate can have both positive and negative effects on brain physiology, including both neuroprotective and neurotoxic effects, and can have both potentiating or inhibiting effects on learning and memory (see [59] for a review). Therefore, the absence of effect of the high-protein modulator may appear less surprising, especially as the mice were given this diet as a small addition to their daily food intake which always consisted of normal laboratory chow present in the home cage.

Additionally, it was shown that in mice, gastric emptying takes around 90 min and gastrointestinal emptying about 3 h [60], at which time the behavioral tasks were mostly finished (as they lasted a maximum of 2 h). Moreover, protein, fats, and carbohydrates have been shown to have different effects on the speed of gastric emptying while also having different rates of absorption into the bloodstream [61]. While sugars and carbohydrates have been shown to slow down gastric emptying [62], they may also have a faster rate of absorption (depending on the form); thus, it is not easy to link rates of absorption to a direct effect on cognition and behavior, as many other factors come into play.

The absence of significant effects of any of the dietary modulators on physical activity may be surprising. However, two important factors need to be borne in mind. First, we were evaluating motor function effects in the context of ‘casual’ exercise and not on the physical performance of highly trained mice (or human athletes) for whom the effect of acute dietary intake pretraining or pre-competition sessions has been extensively evaluated [63]. Our data, therefore, suggest that the motivation to exercise was not altered and thus that a high-fat modulator had distinct effects on behaviors motivated by an external reward (e.g., screen touches in the PR task) versus internally rewarding behaviors, such as voluntary wheel running. Our results also imply that a high-carbohydrate, high-protein, or high-fat snack or drink prior to exercise will not significantly affect performance in the recreational sportsman or woman but that a protein-rich modulator could result in a slight improvement in motor performance. This could be because of positive effects on glucose metabolism, as shown in diabetic patients with a high-protein snack [64], although a chronic high-fat diet could also have been expected to result in reduced blood glucose variations [65]. Second, it is important to distinguish the data we presented here from the effects of a longer-term dietary pattern on physical performance, which was not the aim of our study.

In summary, we were able to demonstrate that mouse cognitive assays using specific time windows of behavioral testing are sensitive enough to successfully detect the short-term effects of different macronutrient ‘snacks’ on cognitive flexibility and motivation. The task-dependent findings are of particular interest given the increasing demand for such snacks and drinks for increasing performance in different intellectually or physically demanding tasks.

## Figures and Tables

**Table 1:** Composition of the different dietary modulators.

**Control Diet**					**Carbohydrate Diet**			
	**Amount (g)**	**Composition**			**Amount (g)**	**Composition**		
Protein	18.5	Egg protein (0.93 g)	Milk protein (17.57 g)		7	Egg protein (0.35 g)	Milk protein (6.65 g)	
Fat	4.5	Butter (2.25 g)	Vegetable oil (2.25 g)		2.6	Butter (1.30 g)	Vegetable oil (1.30 g)	
Carbohydrate	54.2	Glucose (8.67 g)	Starch (8.67 g)	Maltodextrin (36.85 g)	69.7	Glucose (15.47 g)	Starch (15.47 g)	Maltodextrin (38.76 g)
Fiber	4.5	Apple fiber			4.5	Apple fiber		
Minerals (Ash)	6.3	Ca, Fe, P, Mn, Mg, K	Vitamin D3		6.3	Ca, Fe, P, Mn, Mg, K	Vitamin D3	
Water	36				30			
**Protein Diet**					**Fat Diet**			
	**Amount (g)**	**Composition**			**Amount (g)**	**Composition**		
Protein	43.6	Egg protein (2.18 g)	Milk protein (41.42 g)		21.8	Egg protein (1.10 g)	Milk protein (20.70 g)	
Fat	4.5	Butter (2.25 g)	Vegetable oil (2.25 g)		23.2	Butter (11.60 g)	Vegetable oil (11.60 g)	
Carbohydrate	28.9	Glucose (4.62 g)	Starch (4.62 g)	Maltodextrin (19.65 g)	9	Glucose (1.44 g)	Starch (1.44 g)	Maltodextrin (6.12 g)
Fiber	4.5	Apple fiber			4.5	Apple fiber		
Minerals (Ash)	6.3	Ca, Fe, P, Mn, Mg, K	Vitamin D3		6.3	Ca, Fe, P, Mn, Mg, K	Vitamin D3	
Water	35.4				35.2			

## Data Availability

The data presented in this study are available on request from the corresponding author.

## References

[1] Schulze M.B., Martínez-González M.A., Fung T.T., Lichtenstein A.H., Forouhi N.G. (2018). Food based dietary patterns and chronic disease prevention. BMJ.

[2] Bidwell L.C., McClernon F.J., Kollins S.H. (2011). Cognitive enhancers for the treatment of ADHD. Pharmacol. Biochem. Behav..

[3] Hasler C.M. (1996). Functional foods: The western perspective. Nutr. Rev..

[4] Froiland K., Koszewski W., Hingst J., Kopecky L. (2004). Nutritional supplement use among college athletes and their sources of information. Int. J. Sport Nutr. Exerc. Metab..

[5] Kårlund A., Gómez-Gallego C., Turpeinen A.M., Palo-Oja O.-M., El-Nezami H., Kolehmainen M. (2019). Protein Supplements and Their Relation with Nutrition, Microbiota Composition and Health: Is More Protein Always Better for Sportspeople?. Nutrients.

[6] Marinangeli C.P.F., Jones P.J.H. (2013). Gazing into the crystal ball: Future considerations for ensuring sustained growth of the functional food and nutraceutical marketplace. Nutr. Res. Rev..

[7] Vergari F., Tibuzzi A., Basile G. (2010). An overview of the functional food market: From marketing issues and commercial players to future demand from life in space. Adv. Exp. Med. Biol..

[8] Romberg C., Horner A.E., Bussey T.J., Saksida L.M. (2013). A touch screen-automated cognitive test battery reveals impaired attention, memory abnormalities, and increased response inhibition in the TgCRND8 mouse model of Alzheimer’s disease. Neurobiol. Aging.

[9] Urai A.E., Aguillon-Rodriguez V., Laranjeira I.C., Cazettes F., Mainen Z.F., Churchland A.K. (2021). Citric Acid Water as an Alternative to Water Restriction for High-Yield Mouse Behavior. Eneuro.

[10] Reinagel P. (2018). Training Rats Using Water Rewards without Water Restriction. Front. Behav. Neurosci..

[11] Watson P., Beatey S., Wagner F., Stahl T. (1986). Water adulteration with citric acid: Effects on drinking and responsivity to regulatory challenges. Physiol. Behav..

[12] Fischer K., Colombani P.C., Langhans W., Wenk C. (2002). Carbohydrate to protein ratio in food and cognitive performance in the morning. Physiol. Behav..

[13] Romberg C., Yang S., Melani R., Andrews M.R., Horner A.E., Spillantini M.G., Bussey T.J., Fawcett J.W., Pizzorusso T., Saksida L.M. (2013). Depletion of perineuronal nets enhances recognition memory and long-term depression in the perirhinal cortex. J. Neurosci..

[14] Cardinal R.N., Aitken M.R.F. (2010). Whisker: A client–server high-performance multimedia research control system. Behav. Res. Methods.

[15] Markou A., Salamone J.D., Bussey T.J., Mar A.C., Brunner D., Gilmour G., Balsam P. (2013). Measuring reinforcement learning and motivation constructs in experimental animals: Relevance to the negative symptoms of schizophrenia. Neurosci. Biobehav. Rev..

[16] Eagle D.M., Humby T., Dunnett S.B., Robbins T.W. (1999). Effects of regional striatal lesions on motor, motivational, and executive aspects of progressive-ratio performance in rats. Behav. Neurosci..

[17] Hutsell B.A., Newland M.C. (2013). A quantitative analysis of the effects of qualitatively different reinforcers on fixed ratio responding in inbred strains of mice. Neurobiol. Learn. Mem..

[18] van Schaik C.P., Burkart J.M. (2011). Social learning and evolution: The cultural intelligence hypothesis. Philos. Trans. R. Soc. B Biol. Sci..

[19] Izquierdo A., Brigman J., Radke A., Rudebeck P., Holmes A. (2017). The neural basis of reversal learning: An updated perspective. Neuroscience.

[20] Jakobsen M.U., Overvad K. (2011). Macronutrient advice for ischemic heart disease prevention. Curr. Opin. Lipidol..

[21] Alhazmi A., Stojanovski E., McEvoy M., Garg M.L. (2014). Macronutrient intake and type 2 diabetes risk in middle-aged Australian women. Results from the Australian Longitudinal Study on Women’s Health. Public Health Nutr..

[22] Sarris J., Logan A.C., Akbaraly T.N., Amminger G.P., Balanzá-Martínez V., Freeman M.P., Hibbeln J., Matsuoka Y., Mischoulon D., Mizoue T. (2015). Nutritional medicine as mainstream in psychiatry. Lancet Psychiatry.

[23] Solfrizzi V., Custodero C., Lozupone M., Imbimbo B.P., Valiani V., Agosti P., Schilardi A., D’Introno A., La Montagna M., Calvani M. (2017). Relationships of dietary patterns, foods, and micro-and macronutrients with Alzheimer’s disease and late-life cognitive disorders: A systematic review. J. Alzheimer’s Dis..

[24] Roberts R.O., Roberts L.A., Geda Y.E., Cha R.H., Pankratz V.S., O’Connor H.M., Knopman D.S., Petersen R.C. (2012). Relative intake of macronutrients impacts risk of mild cognitive impairment or dementia. J. Alzheimer’s Dis..

[25] Dye L., Lluch A., Blundell J.E. (2000). Macronutrients and mental performance. Nutrition.

[26] Brigman J.L., Graybeal C., Holmes A. (2010). Predictably irrational: Assaying cognitive inflexibility in mouse models of schizophrenia. Front. Neurosci..

[27] McGonigle P., Ruggeri B. (2014). Animal models of human disease: Challenges in enabling translation. Biochem. Pharmacol..

[28] Nithianantharajah J., McKechanie A.G., Stewart T.J., Johnstone M., Blackwood D.H., Clair D.S., Grant S.G.N., Bussey T.J., Saksida L.M. (2015). Bridging the translational divide: Identical cognitive touchscreen testing in mice and humans carrying mutations in a disease-relevant homologous gene. Sci. Rep..

[29] Bussey T., Holmes A., Lyon L., Mar A., McAllister K., Nithianantharajah J., Oomen C., Saksida L. (2012). New translational assays for preclinical modelling of cognition in schizophrenia: The touchscreen testing method for mice and rats. Neuropharmacology.

[30] Horner A.E., Heath C.J., Hvoslef-Eide M., Kent B.A., Kim C.H., Nilsson S.R., Alsiö J., Oomen C.A., Holmes A., Saksida L.M. (2013). The touchscreen operant platform for testing learning and memory in rats and mice. Nat. Protoc..

[31] Robbins T., James M., Owen A., Sahakian B., McInnes L., Rabbitt P. (1994). Cambridge Neuropsychological Test Automated Battery (CANTAB): A factor analytic study of a large sample of normal elderly volunteers. Dementia.

[32] Auersperg A.M., Gajdon G.K., von Bayern A.M. (2012). A new approach to comparing problem solving, flexibility and innovation. Commun. Integr. Biol..

[33] Haier R.J., Siegel B.V., MacLachlan A., Soderling E., Lottenberg S., Buchsbaum M.S. (1992). Regional glucose metabolic changes after learning a complex visuospatial/motor task: A positron emission tomographic study. Brain Res..

[34] Jonides J., Schumacher E.H., Smith E.E., Lauber E.J., Awh E., Minoshima S., Koeppe R.A. (1997). Verbal working memory load affects regional brain activation as measured by PET. J. Cogn. Neurosci..

[35] Haier R.J., Siegel B., Tang C., Abel L., Buchsbaum M.S. (1992). Intelligence and changes in regional cerebral glucose metabolic rate following learning. Intelligence.

[36] Kennedy D., Scholey A. (2000). Glucose administration, heart rate and cognitive performance: Effects of increasing mental effort. Psychopharmacology.

[37] Wu A., Ying Z., Gomez-Pinilla F. (2004). Dietary omega-3 fatty acids normalize BDNF levels, reduce oxidative damage, and counteract learning disability after traumatic brain injury in rats. J. Neurotrauma.

[38] Wu A., Molteni R., Ying Z., Gomez-Pinilla F. (2003). A saturated-fat diet aggravates the outcome of traumatic brain injury on hippocampal plasticity and cognitive function by reducing brain-derived neurotrophic factor. Neuroscience.

[39] McCann J.C., Ames B.N. (2005). Is docosahexaenoic acid, an n-3 long-chain polyunsaturated fatty acid, required for development of normal brain function? An overview of evidence from cognitive and behavioral tests in humans and animals. Am. J. Clin. Nutr..

[40] Suzuki H., Park S.J., Tamura M., Ando S. (1998). Effect of the long-term feeding of dietary lipids on the learning ability, fatty acid composition of brain stem phospholipids and synaptic membrane fluidity in adult mice: A comparison of sardine oil diet with palm oil diet. Mech. Ageing Dev..

[41] Wu A., Ying Z., Gomez-Pinilla F., Guley N.H., Rogers J.T., Del Mar N.A., Deng Y., Islam R.M., D’Surney L., Ferrell J. (2007). Omega-3 fatty acids supplementation restores mechanisms that maintain brain homeostasis in traumatic brain injury. J. Neurotrauma.

[42] Winocur G., Greenwood C.E. (2005). Studies of the effects of high fat diets on cognitive function in a rat model. Neurobiol. Aging.

[43] Molteni R., Barnard R., Ying Z., Roberts C., Gómez-Pinilla F. (2002). A high-fat, refined sugar diet reduces hippocampal brain-derived neurotrophic factor, neuronal plasticity, and learning. Neuroscience.

[44] Calvo-Ochoa E., Hernández-Ortega K., Ferrera P., Morimoto S., Arias C. (2014). Short-term high-fat-and-fructose feeding produces insulin signaling alterations accompanied by neurite and synaptic reduction and astroglial activation in the rat hippocampus. J. Cereb. Blood Flow Metab..

[45] Parrott M.D., Greenwood C.E. (2007). Dietary influences on cognitive function with aging: From high-fat diets to healthful eating. Ann. New York Acad. Sci..

[46] Tran D.M., Westbrook R.F. (2017). A high-fat high-sugar diet-induced impairment in place-recognition memory is reversible and training-dependent. Appetite.

[47] Beilharz J., Maniam J., Morris M. (2016). Short-term exposure to a diet high in fat and sugar, or liquid sugar, selectively impairs hippocampal-dependent memory, with differential impacts on inflammation. Behav. Brain Res..

[48] Boitard C., Cavaroc A., Sauvant J., Aubert A., Castanon N., Layé S., Ferreira G. (2014). Impairment of hippocampal-dependent memory induced by juvenile high-fat diet intake is associated with enhanced hippocampal inflammation in rats. Brain Behav. Immun..

[49] Holloway C.J., Cochlin L.E., Emmanuel Y., Murray A., Codreanu I., Edwards L.M., Szmigielski C., Tyler D.J., Knight N.S., Saxby B.K. (2011). A high-fat diet impairs cardiac high-energy phosphate metabolism and cognitive function in healthy human subjects. Am. J. Clin. Nutr..

[50] Forouhi N.G., Krauss R.M., Taubes G., Willett W. (2018). Dietary fat and cardiometabolic health: Evidence, controversies, and consensus for guidance. BMJ.

[51] Lutas A., Yellen G. (2013). The ketogenic diet: Metabolic influences on brain excitability and epilepsy. Trends Neurosci..

[52] Hallböök T., Ji S., Maudsley S., Martin B. (2011). The effects of the ketogenic diet on behavior and cognition. Epilepsy Res..

[53] Betz A.L., Goldstein G., Katzman R., Siegel G.J. (1994). Blood-brain-cerebrospinal fluid barriers. Basic Neurochemistry: Molecular, Cellular, and Medical Aspects.

[54] Institute of Medicine (US) Committee on Military Nutrition Research (1999). 14. Amino Acid and Protein Requirements: Cognitive Performance, Stress, and Brain Function. The Role of Protein and Amino Acids in Sustaining and Enhancing Performance.

[55] Marriott B.M., Institute of Medicine (US) Committee on Military Nutrition Research (1994). 15. Tyrosine and Stress: Human and Animal Studies. Food Components to Enhance Performance: An Evaluation of Potential Performance-Enhancing Food Components for Operational Rations.

[56] Klein M.O., Battagello D.S., Cardoso A.R., Hauser D.N., Bittencourt J.C., Correa R.G. (2018). Dopamine: Functions, Signaling, and Association with Neurological Diseases. Cell. Mol. Neurobiol..

[57] Fernstrom M.H., Fernstrom J.D. (1995). Brain tryptophan concentrations and serotonin synthesis remain responsive to food consumption after the ingestion of sequential meals. Am. J. Clin. Nutr..

[58] Lieberman H.R., Caballero B., Finer N. (1986). The composition of lunch determines afternoon plasma tryptophan ratios in humans. J. Neural. Transm..

[59] Onaolapo A.Y., Onaolapo O.J. (2020). Dietary glutamate and the brain: In the footprints of a Jekyll and Hyde molecule. Neurotoxicology.

[60] Schwarz R., Kaspar A., Seelig J., Künnecke B. (2002). Gastrointestinal transit times in mice and humans measured with 27Al and 19F nuclear magnetic resonance. Magn. Reson. Med..

[61] Carreiro A.L., Dhillon J., Gordon S., Higgins K.A., Jacobs A.G., McArthur B.M., Redan B.W., Rivera R.L., Schmidt L.R., Mattes R.D. (2016). The Macronutrients, Appetite, and Energy Intake. Annu. Rev. Nutr..

[62] Marciani L., Cox E.F., Pritchard S.E., Major G., Hoad C.L., Mellows M., Hussein M.O., Costigan C., Fox M., Gowland P.A. (2015). Additive effects of gastric volumes and macronutrient composition on the sensation of postprandial fullness in humans. Eur. J. Clin. Nutr..

[63] Ormsbee M.J., Bach C.W., Baur D.A. (2014). Pre-Exercise Nutrition: The Role of Macronutrients, Modified Starches and Supplements on Metabolism and Endurance Performance. Nutrients.

[64] Yang J., Park H.J., Hwang W., Kim T.H., Kim H., Oh J., Cho M.S. (2021). Changes in the glucose and insulin responses according to high-protein snacks for diabetic patients. Nutr. Res. Pract..

[65] Imamura F., Micha R., Wu J.H.Y., de Oliveira Otto M.C., Otite F.O., Abioye A.I., Mozaffarian D. (2016). Effects of Saturated Fat, Polyunsaturated Fat, Monounsaturated Fat, and Carbohydrate on Glucose-Insulin Homeostasis: A Systematic Review and Meta-analysis of Randomised Controlled Feeding Trials. PLoS Med..

